# Administration route governs the therapeutic efficacy, biodistribution and macrophage targeting of anti-inflammatory nanoparticles in the lung

**DOI:** 10.1186/s12951-021-00803-w

**Published:** 2021-02-25

**Authors:** Lu Wang, Yafei Rao, Xiali Liu, Liya Sun, Jiameng Gong, Huasheng Zhang, Lei Shen, Aihua Bao, Hong Yang

**Affiliations:** 1 Department of Pulmonary and Critical Care Medicine, Shanghai General Hospital, Shanghai Jiao Tong University School of Medicine, Shanghai, 201620 China; 2 School of Biomedical Engineering, Tianjin Medical University, Tianjin, 300070 China; 3 Shanghai Institute of Immunology, Shanghai Jiaotong University School of Medicine, Shanghai, 200025 China; 4 The Province and Ministry Co-Sponsored Collaborative Innovation Center for Medical Epigenetics, Tianjin Medical University, Tianjin, 300070 China; 5 Present Address: Department of Critical Care Medicine, Zhongda Hospital, School of Medicine, Southeast University, Nanjing, 210009 China

**Keywords:** Nanoparticle, Administration route, Bio-distribution, Acute lung injury, Inflammation, Pulmonary macrophage

## Abstract

**Background:**

Uncontrolled inflammation is a central problem for many respiratory diseases. The development of potent, targeted anti-inflammatory therapies to reduce lung inflammation and re-establish the homeostasis in the respiratory tract is still a challenge. Previously, we developed a unique anti-inflammatory nanodrug, P12 (made of hexapeptides and gold nanoparticles), which can attenuate Toll-like receptor-mediated inflammatory responses in macrophages. However, the effect of the administration route on its therapeutic efficacy and tissue distribution remained to be defined.

**Results:**

In this study, we systematically compared the effects of three different administration routes [the intratracheal (i.t.), intravenous (i.v.) and intraperitoneal (i.p.)] on the therapeutic activity, biodistribution and pulmonary cell targeting features of P12. Using the LPS-induced ALI mouse model, we found that the local administration route via i.t. instillation was superior in reducing lung inflammation than the other two routes even treated with a lower concentration of P12. Further studies on nanoparticle biodistribution showed that the i.t. administration led to more accumulation of P12 in the lungs but less in the liver and other organs; however, the i.v. and i.p. administration resulted in more nanoparticle accumulation in the liver and lymph nodes, respectively, but less in the lungs. Such a lung favorable distribution was also determined by the unique surface chemistry of P12. Furthermore, the inflammatory condition in the lung could decrease the accumulation of nanoparticles in the lung and liver, while increasing their distribution in the spleen and heart. Interestingly, the i.t. administration route helped the nanoparticles specifically target the lung macrophages, whereas the other two administration routes did not.

**Conclusion:**

The i.t. administration is better for treating ALI using nanodevices as it enhances the bioavailability and efficacy of the nanodrugs in the target cells of the lung and reduces the potential systematic side effects.
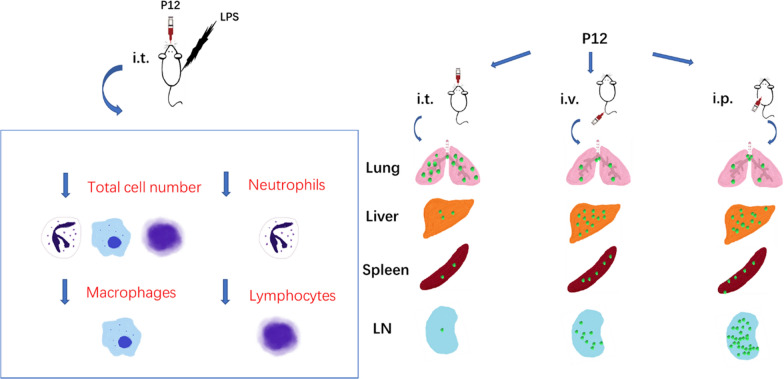

## Introduction

Lung is a unique organ that is continuously exposed to the outside environment. During each breath for gas exchanging in the lung, the respiratory tract constantly senses and responds to various stimuli from the outside environment, such as allergens (e.g., dust mites and pollens), pollutants (e.g., PM2.5), pathogens (virus, fungus and bacteria) and toxic gases [1, 2]. Thus, it is very important to regulate such reactions to maintain the homeostasis in the lung. When the balance is shifted toward pathogenesis, many chronic and acute lung diseases occur, including asthma, chronic obstructive pulmonary disease (COPD), lung cancer, interstitial pulmonary fibrosis and acute respiratory distress syndrome (ARDS, e.g. COVID-19) [3–6]. A common feature for these respiratory disorders is the uncontrolled inflammatory reaction in the lung [7, 8]. Unfortunately, there is still a lack of effective and safe clinical treatments to manage the overwhelming inflammation in the lung. Although the widely used anti-inflammatory drugs glucocorticoids can reduce inflammation locally and systematically, the long-term use of glucocorticoids is often associated with serious side effects [9–11]. Therefore, there is an urgent need for a potent, targeted anti-inflammatory therapy with minimum side effect to promote the tissue repair and re-establish the homeostasis (i.e., immune balance) in the respiratory tract.

Targeted therapy represents a new promising treatment modality to precisely act on specific cells/tissues or key molecular pathways to reduce adverse effects resulting from less targeted treatments. It can be achieved by either drug design specific to the target or targeted delivery method. In the latter case, nanomaterials provide prominent advantages for targeted delivery of drug compounds as they have prolonged circulation time, preferred cells/tissues accumulation (e.g., tumor) and desired bioavailability by tunable functionality. However, emerging evidence has shown that almost all nanodevices tend to accumulate and remain in the reticuloendothelial system (RES) organs (i.e., liver and spleen); this in turn could reduce the bioavailability and increase certain organ toxicity of the drugs [12, 13]. It has been found that less than 0.7% of nanodrugs were actually accumulated in tumor sites regardless an active targeting motif was employed [14]. Therefore, how to obtain favorable organ distribution for a given nanodevice is still a challenge in the field of nano-delivery.

In the case of pulmonary diseases, lung is a unique organ, allowing the administration of drugs directly into the airway via inhalation or intratracheal (i.t.) instillation to achieve local delivery. On the other hand, lungs consist of a large blood vessel network for gas exchange, and thus can also be reached systematically through the circulation system. Although rarely used clinically, intraperitoneal (i.p.) administration is often conducted experimentally for its convenience, which can also allow the adsorption of nanodrugs into the circulation and then to the lung. Considering the clinical relevance, intravenous (i.v.) administration is favorably chosen for most of the reported therapeutic nanoparticles for anti-cancer treatments. However, the comparison among different routes of administration on their therapeutic efficacy, biodistribution, excretion and cell targeting efficiency in vivo are required in order to choose the best one for a specific clinical application. Therefore, we set out this study to investigate the effect of different administration routes on the efficacy and biodistribution of nanodrugs to treat lung diseases.

Previously, we developed a unique nano-system that is made of hexapeptides and gold nanoparticles (GNPs), designated as P12 [15]. Interestingly, neither the hexapeptide nor the GNP alone can regulate Toll-like receptor (TLR) signaling pathways; however, when they formed the hybrid P12, it was capable of attenuating multiple TLR signaling pathways and the down-stream inflammatory reactions in macrophages [16, 17]. P12 can also ameliorate lung injury and inflammation in the lipopolysaccharide (LPS)-induced acute lung injury (ALI) mouse model by targeting the lung macrophages and promoting their differentiation into an anti-inflammatory M2 phenotype [18]. A fluorescent probe can be conjugated to P12 for visualizing the distribution of these nanoparticles in vivo [19], while the gold core of P12 can be quantified by the inductively coupled plasma mass spectrometry (ICP-MS) at minuscule levels. These properties make P12 an ideal system to study the therapeutic efficiency and biodistribution of nano-devices in an in vivo system.

Herein, we aimed to identify the optimal administration route of P12 in managing lung inflammation with better therapeutic efficiency, preferential biodistribution and effective cell targeting. Using a classical LPS-induced ALI mouse model, we first investigated the effects of three different administration routes (i.t., i.v. and i.p.) on the therapeutic efficacy of P12 by assessing the lung inflammation and injury levels. We then analyzed the biodistribution profiles of P12 in the healthy and ALI mice using both the ICP-MS and ex vivo imaging techniques. Finally, we evaluated the in vivo targeting efficiency of P12 to the lung macrophages among the three administration routes. This work helps better understand the biodistribution and effectiveness of nanoparticles in the respiratory tract under different administration routes, which is critical for the rational design of nano-delivery systems for respiratory diseases. It will also facilitate the development of effective targeted nano-therapies to treat ALI/ARDS.

## Materials and methods

### Materials

Tetrachloroauric acid trihydrate (99.9%), LPS (E-coli O111:B4), DNase I and collagenase II were purchased from Sigma (Sant-Louis, MO, USA). Hexapeptide was synthesized from Nanjing Jietai Biological Company (Nanjing, China). The heterobifunctional polyethylene glycol (PEG) derivatives, including Cy5 labeled PEG thiol (HS-PEG5000-Cy5), methoxy PEG thiol (HS-PEG2000-CH_3_) and hydroxyl PEG thiol (HS-PEG2000-OH) were purchased from Ponsure Biotechnology (Shanghai, China). Murine GM-CSF was from PeproTech (Rocky Hill, NJ, USA). Mouse IL-6 and MCP-1 ELISA kits were purchased from Invitrogen (Grand Island, NY, USA). Liu stain was purchased from Baso Diagnostics Inc. (Zhuhai, China). RBC Lysis Buffer was obtained from Beyotime (Shanghai, China). Fluorochrome-conjugated antibodies against mouse F4/80 (123110) and CD11b (101215) were from BioLegend (San Diego, CA, USA), while Fc Block (562681) and Fluorochrome-conjugated antibodies against mouse CD45 (563410), CD19 (557655), CD3 (563024), CD11c (562782) and Gr1 (553126) were from BD Biosciences (San Diego, CA, USA). Viability dye (L34962) was purchased from Life Technologies (Carlsbad, CA, USA).

### Nanoparticle formulation and characterization

Gold nanoparticles (GNPs, diameter 13 nm) were synthesized using the method according to the literature and our earlier research [16, 20]. The fabrication of hexapeptide modified GNPs (P12) was carried out based on our previous publication [15, 16]. Briefly, the hexapeptide (CLPFFD) stock solution (1 mM) was prepared in ultrapure water. Ten volumes of the GNP solution were mixed with one volume of the peptide stock solution and incubated overnight to form P12. The Cy5 labeled P12 (P12-Cy5) was made the same way where the peptide stock solution contained 1% HS-PEG5000-Cy5 (1 mM). All the peptide-GNP hybrids solution was sterilized by filtering through a syringe filter (0.22 µm, Millipore, Billerica, MA, USA), followed by washing three times with sterile phosphate buffered saline (PBS) to remove the unbound peptide ligands by centrifugation (15,000 rpm at 4℃ for 30 min). The hybrids were resuspended in PBS or cell culture medium to a final concentration of 125 nM or 500 nM before use.

The bare GNPs and P12 were characterized by JEOL JEM-2100 transmission electron microscope (Tokyo, Japan) and dynamic light scattering (DLS) (Malvern Instruments, Worcestershire, UK) for their morphology and size. Their stability was assessed by mixing with equal volume of various concentrations of NaCl solution in a 96-well plate for 2 h at room temperature, and then measuring the optical density (OD) of GNPs at 524 nm on a microplate reader (TECAN, Männedorf, Switzerland). The data were normalized with the OD value of the mixtures in water.

### Bone marrow-derived macrophage culture and cytokine analysis

Bone marrow cells were flushed from femurs and tibias of C57BL/6 J wild-type mice as previously described [21]. The cells were cultured in IMDM medium containing 10% FBS and GM-CSF (20 ng/mL) for 7 days. Macrophages were harvested on day 7 and plated at a density of 1 × 10^5^ cells/well in a 96-well plate. The adherent macrophages were stimulated with 10 ng/mL LPS alone or together with P12 for 24 h; the culture media were then collected for cytokine analysis (IL-6 and MCP-1) by ELISA kits following the manufacturer’s protocols.

### LPS-induced acute lung injury (ALI) mouse model

Male C57BL/6 J wild-type mice (6–8 weeks) were purchased from Shanghai SLAC Laboratory Animal Co. Ltd. (Shanghai, China). Mice were kept in the animal experimental center of Shanghai General Hospital with specific-pathogen-free conditions. All animal experiments were reviewed and approved by the Institutional Animal Care and Use Committee of Shanghai General Hospital (Approval number # 2018KY201).

Mice were anesthetized by intraperitoneal (i.p.) injection of 1% sodium pentobarbital (45 mg/kg), followed by intratracheal injection of LPS (10 mg/kg) as previously described [18]. To study the therapeutic effects of P12 on ALI through different administration routes, mice were pretreated with P12 (500 nM or 125 nM in 50 μL PBS) via intratracheal (i.t.), intravenous (i.v.) and i.p. injection 2 h prior to LPS challenge. The control groups were given the same volume of PBS with the same administration route as P12. Mice were sacrificed 24 h after LPS challenge to assess lung inflammation and injury.

### Bronchoalveolar lavage fluid (BALF) collection and differential cell counting

The bronchoalveolar lavage (BAL) was performed by injecting 0.8 mL of ice-cold PBS twice into the lung through tracheal incision. The collected BALF was centrifuged at 1000 rpm for 10 min at 4℃. The cell pellets were treated with glacial acetic acid (3% in PBS) to remove red blood cells and were resuspended in PBS for total cell counts using a hemocytometer. Aliquots of cell suspensions (about 1 × 10^4^ cells) were spun onto a glass slide using cytospin (StatSpin, USA), followed by Liu staining. The differential cell counts were carried out by counting more than 200 cells under an optical microscope.

### Lung histology analysis

In a separate group of mice, the left lungs were harvested without BAL performance and fixed in 4% paraformaldehyde at room temperature. The fixed lungs were embedded in paraffin to obtain 5 μm thick sections, which were stained with hematoxylin and eosin (H&E) and imaged on a Leica Microsystem. The images (at least five random 200 × fields per lung) were independently and blindly scored by two researchers according to the international scoring standard of the following five parameters: (i) neutrophils in the alveolar space, (ii) neutrophils in the interstitial space, (iii) hyaline membranes, (iv) proteinaceous debris filling the airspaces, and (v) alveolar septal thickening [22]. To calculate the total score of the lung injury, the sum of each of these five independent variables were weighted according to their contribution to ALI, and then were normalized to the number of fields. The final injury score is a value between zero and one (inclusive).

### The analysis of P12 levels in different organs/tissues

To visualize P12 in various organs, mice were treated with Cy5-labeled P12 through i.t., i.p. and i.v. injection for 4 h. The major organs/tissues including lung, liver, spleen, heart, kidney, lymph node (LN) and gastrointestinal tract (G.I.T) were collected after sacrificing the mice. Ex vivo fluorescence imaging was conducted (excitation: 620 nm; emission; 670 nm) using the IVIS Lumina II Imaging System (Perkin Elmer, Caliper Life Sciences, Hopkinton, USA). In the ALI mouse model, mice were treated with LPS (10 mg/kg) for 2 h prior to P12-Cy5 administration.

To further quantify the amount of P12 in each organ/tissue, the inductively coupled plasma mass spectrometry (ICP-MS, iCAP Q, Thermo Fisher Scientific, Bremen, Germany) was applied. The dissected organs/tissues were weighed and digested with acids as described previously [19] prior to ICP-MS analysis.

### Flow cytometry analysis and preparation of lung single cell suspensions

After BAL, lungs were cut into small pieces and digested in RPMI-1640 medium containing collagenase II (1 mg/mL) and DNase I (0.15 mg/mL) at 37℃ for 1 h. The digested tissues were prepared into a single cell suspension by vigorously shaking and passing through a 70 μm cell strainer after removing the red blood cells. Cells collected from the BALF and the lung single cell suspensions were first stained with viability dye, and then processed with Fc Block to reduce nonspecific binding; they were subsequently stained with a panel of fluorochrome-conjugated antibodies against F4/80, CD11b, CD45, CD19, CD3, CD11c and Gr1. Flow cytometry was performed using LSR Fortessa X30 (BD Bioscience, USA), and data were analyzed with FlowJo software.

### Statistical analysis

Data were presented as mean ± standard error of mean (SEM). Differences between two groups were analyzed by unpaired t-test, and differences among multiple groups were analyzed by one-way ANOVA with Bonferroni post hoc test using GraphPad Prism 7. The p value less than 0.05 was considered statistically significant.

## Results

### Synthesis and characterization of hexapeptide modified GNPs

**Fig. 1:**
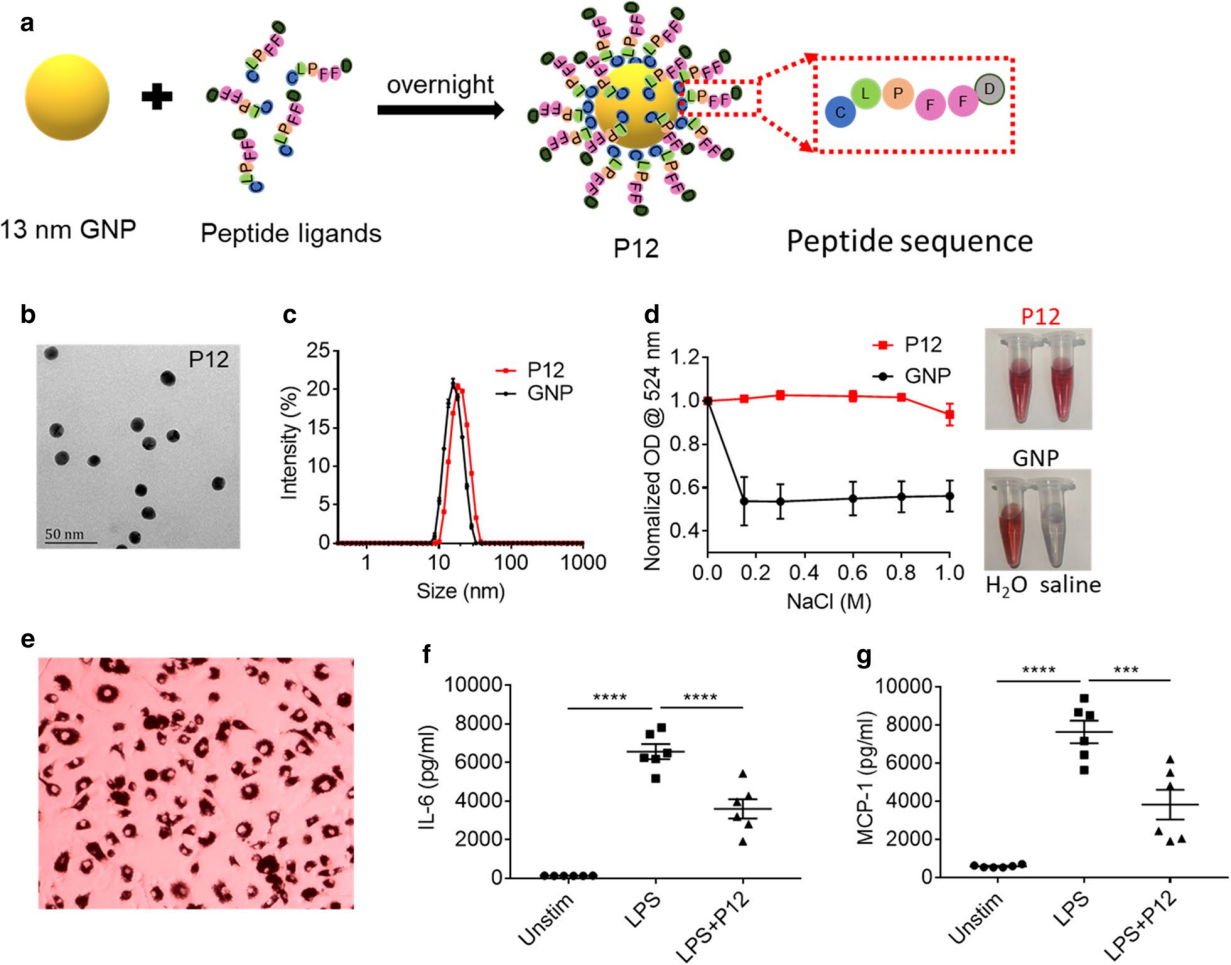
Synthesis and characterization of the anti-inflammatory peptide–GNP hybrid P12. **a** A diagram of the P12 synthesis process. **b** The TEM image of P12. **c** The hydrodynamic size of P12 measured by DLS. **d** The stability of P12 in various concentrations of NaCl (0–1 M) with representative photographs (at 150 mM NaCl) on the right. **e** The optical microscopic image of BMDMs treated with LPS and P12 (× 400). **f**, **g** The levels of pro-inflammatory cytokines IL-6 (**f**) and MCP-1 (**g**) in BMDMs stimulated with LPS (10 ng/mL) for 24 h in the presence and absence of P12 treatment. P12 = 100 nM; ***p < 0.001, ****p < 0.0001

Previously, we developed a novel class of anti-inflammatory peptide–GNP hybrids named P12, which was composed of a 13 nm GNP core and a surface coating of hexapeptides (CLPFFD) (Fig. 1a). Our earlier work demonstrated that i.t. administration of P12 could regulate lung inflammation of LPS-induced ALI mice by inhibiting multiple TLR pathways and promoting the polarization of pulmonary macrophages to M2 phenotype [18, 23].

Under transmission electron microscopy (TEM), P12 showed a uniform spherical structure (Fig. 1b). The hydrodynamic diameter of P12 was slightly larger than that of bare GNP by dynamic light scattering (DLS) analysis (Fig. 1c), confirming the presence of peptides on the GNP surface. In addition, P12 was more stable in salt solution (0.2–1.0 M) than bare GNP (Fig. 1d).

The anti-inflammatory activity of P12 was next verified in murine bone marrow-derived macrophages (BMDMs). After cells were treated with LPS alone or together with P12 for 24 h, we found that a large number of P12 were taken up by BMDMs (Fig. 1e). Through the assessment of the pro-inflammatory cytokine levels, we found that P12 significantly decreased the LPS-induced interleukin-6 (IL-6) and monocyte chemoattractant protein-1 (MCP-1) production (Fig. 1f and g).

### The therapeutic efficacy of P12 on the LPS-induced ALI mouse model through different administration routes

The administration route of a given drug is particularly important for its therapeutic activity in vivo. Although our previous studies have shown that P12 could effectively alleviate the lung inflammation through i.t. injection [18, 19], the question whether the i.t. route is the optimum administration route for nanodrugs to treat lung diseases still remains. To answer this question, we systematically studied the effects of three different administration routes (i.t., i.v. and i.p.) on the therapeutic activity of P12 in the LPS-induced ALI mouse model with two different concentrations of P12.

**Fig. 2:**
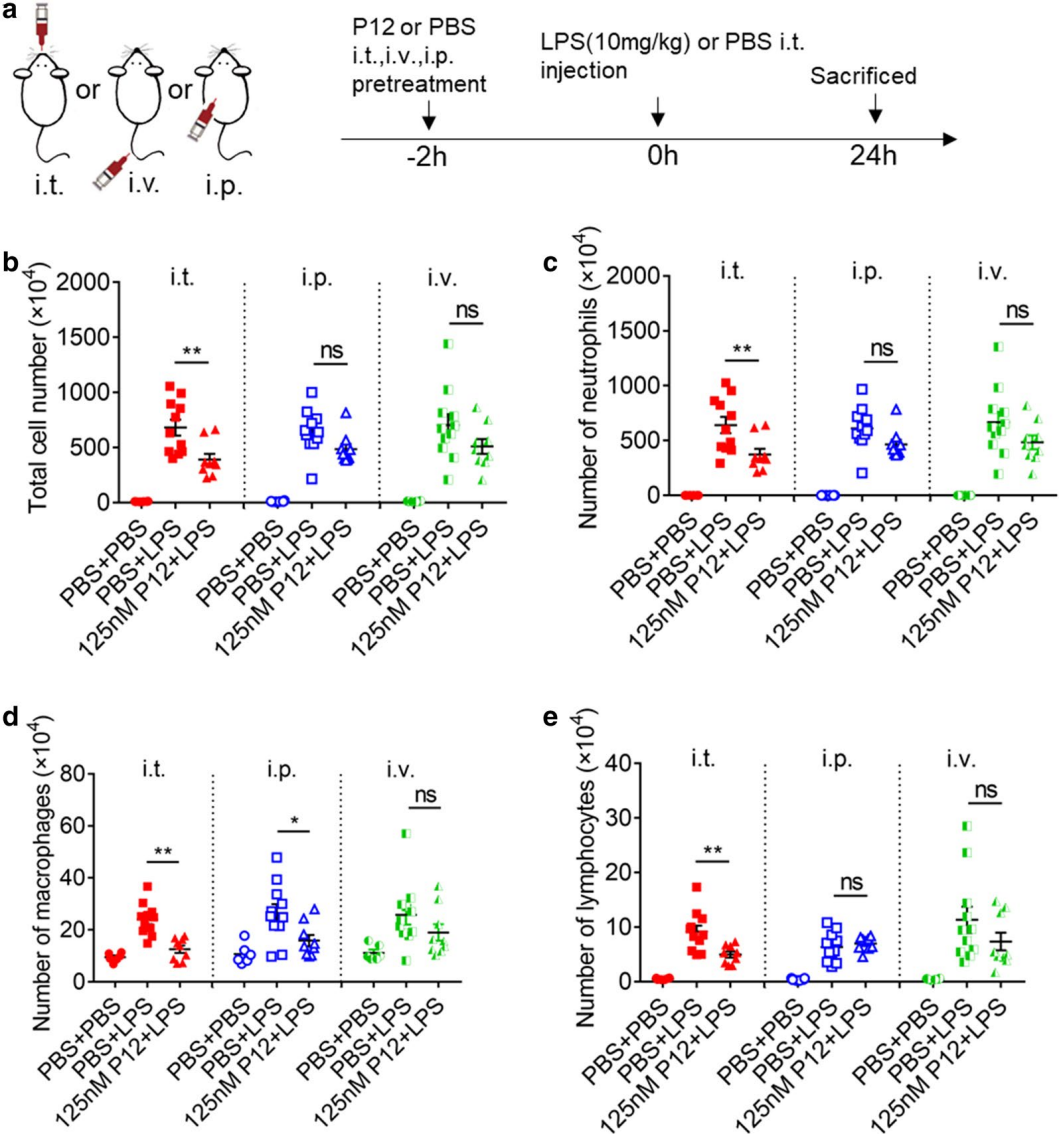
The effect of administration routes on cell infiltration in the BALF in the LPS-induced ALI mouse with a lower dose of P12. **a** A schematic diagram of LPS-induced ALI model with the pretreatment of P12 (50 μL, 125 nM) or the same volume of PBS through i.t., i.v. or i.p. administration 2 h before LPS (10 mg/kg, i.t.) challenge. Mice were sacrificed to harvest the BALF 24 h after LPS injection for the analysis of the number of total cells (**b**), neutrophils (**c**), macrophages (**d**) and lymphocytes (**e**). N ≥ 6 per group, ns: not significant, *p < 0.05, **p < 0.01

**Fig. 3:**
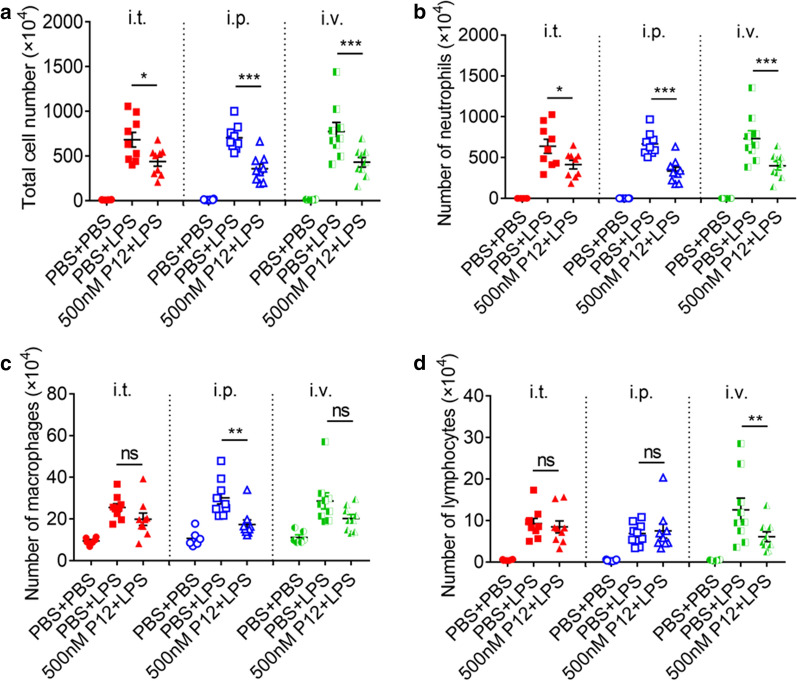
The effect of administration routes on cell infiltration in the BALF in the LPS-induced ALI mouse with a higher dose of P12. The BALF was collected for the analysis of the number of total cells (**a**), neutrophils (**b**), macrophages (**c**) and lymphocytes (**d**). The concentration of P12 = 500 nM. N ≥ 6 per group, ns: not significant, *p < 0.05, ** p < 0.01, *** p < 0.001

As shown in Fig. 2, at a low concentration of P12 (125 nM), only i.t. administration could significantly decrease the number of total cells, neutrophils, macrophages and lymphocytes in the BALF of LPS-induced ALI mice (Fig. 2b–e). Surprisingly, the i.v. administration of P12 had no effects on the reduction of the BALF inflammatory cell infiltration (Fig. 2b–e); the i.p. administration could only decrease the number of BAL macrophages in ALI mice (Fig. 2d). However, when P12 was given at a higher concentration (500 nM), P12 was able to reduce the total cell count and neutrophil count in the BALF of ALI mice regardless of the three different administration routes (Fig. 3). Interestingly, the i.p. and i.v. administration of P12 could still decrease macrophage count and lymphocyte count in the BALF, respectively (Fig. 3c, d), but the i.t. administration of P12 could not.

**Fig. 4:**
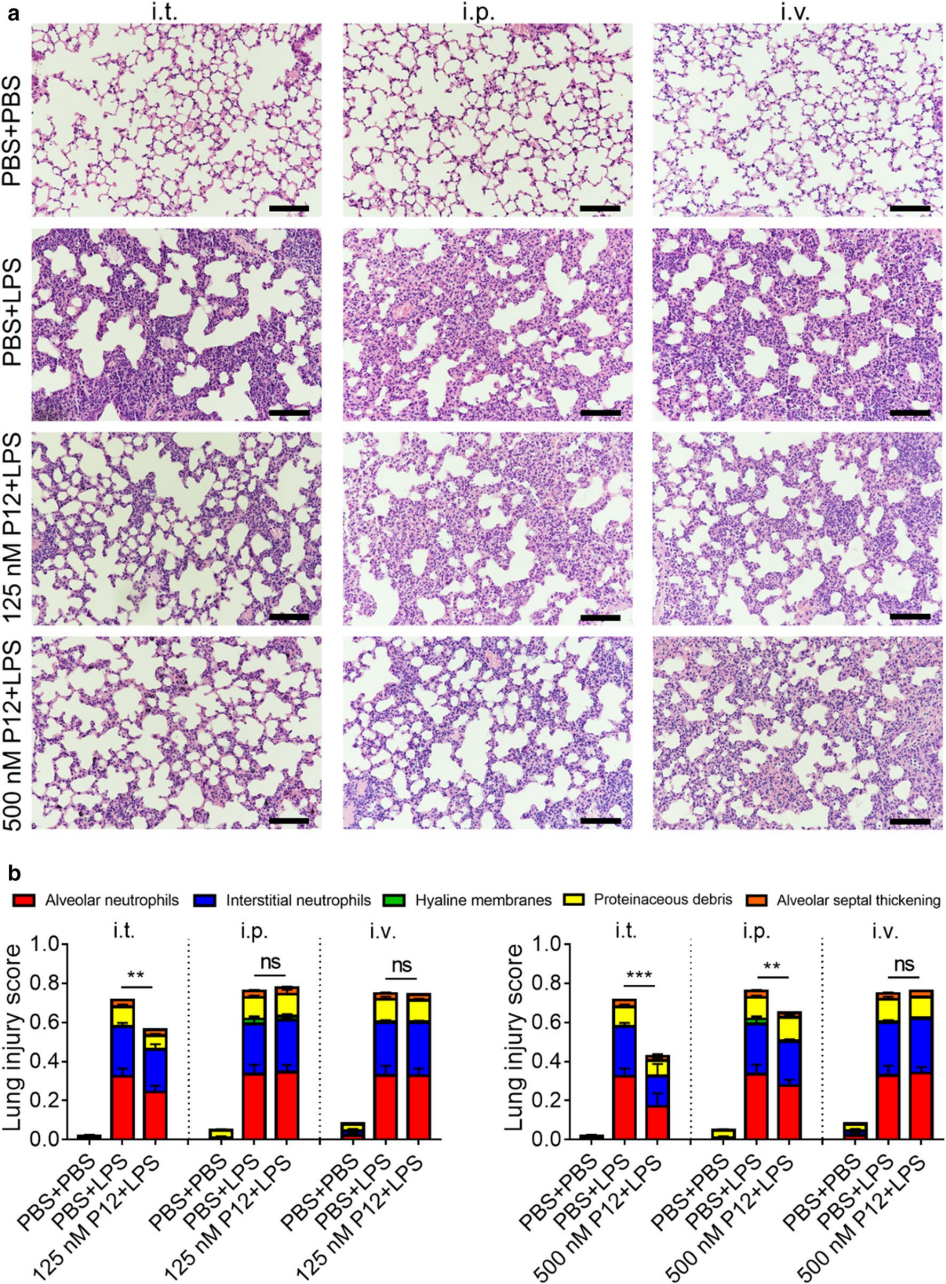
The effect of the administration routes of P12 on the lung inflammation and injury in LPS-induced ALI mice. **a** Representative histological images (× 200) of lung sections with hematoxylin and eosin (H&E) staining from PBS + PBS, PBS + LPS, P12 (125 nM) + LPS and P12 (500 nM) + LPS groups; Mice were pretreated P12 through i.t., i.p. or i.v. administration, followed by intratracheal instillation of LPS. Scale bar = 100 µm. **b** Lung injury scores of the lung sections from each group for two different P12 concentrations (125 nM at left; 500 nM at right). N = 6 per group, ns: not significant, **p < 0.01, ***p < 0.001

The degree of lung injury was assessed with lung tissue histopathology among the three different administration routes. The international scoring standard was applied to analyze the accumulation of neutrophils in the alveolar and interstitial space, formation of hyaline membranes, presence of proteinaceous debris filling the airspaces, and the thickness of the alveolar wall [22]. Comparing to PBS control group (no injury), the LPS challenge exhibited severe acute lung injury. The pretreatment of P12 was effective to reduce the lung injury via i.t. administration regardless of P12 concentration (Fig. 4). Only at a higher concentration of P12 would it be effective to decrease the lung injury score for the i.p. administration route, while P12 given intravenously did not have effect on the lung injury score at both concentrations (Fig. 4b). This observation was similar to the trend found in the inflammatory cell infiltration analysis in the BALF (Figs. 2 and 3).

Collectively, these results demonstrated that the administration route could affect the therapeutic efficacy of P12, and such an effect was nanoparticle concentration dependent. At a higher concentration, P12 exhibited similar therapeutic effectiveness in reducing lung inflammation no matter which administration route was employed; however, at a lower concentration, the local i.t. injection of P12 into the lung was the most effective administration route among the three.

### Biodistribution of P12 in healthy mice through different administration routes

**Fig. 5:**
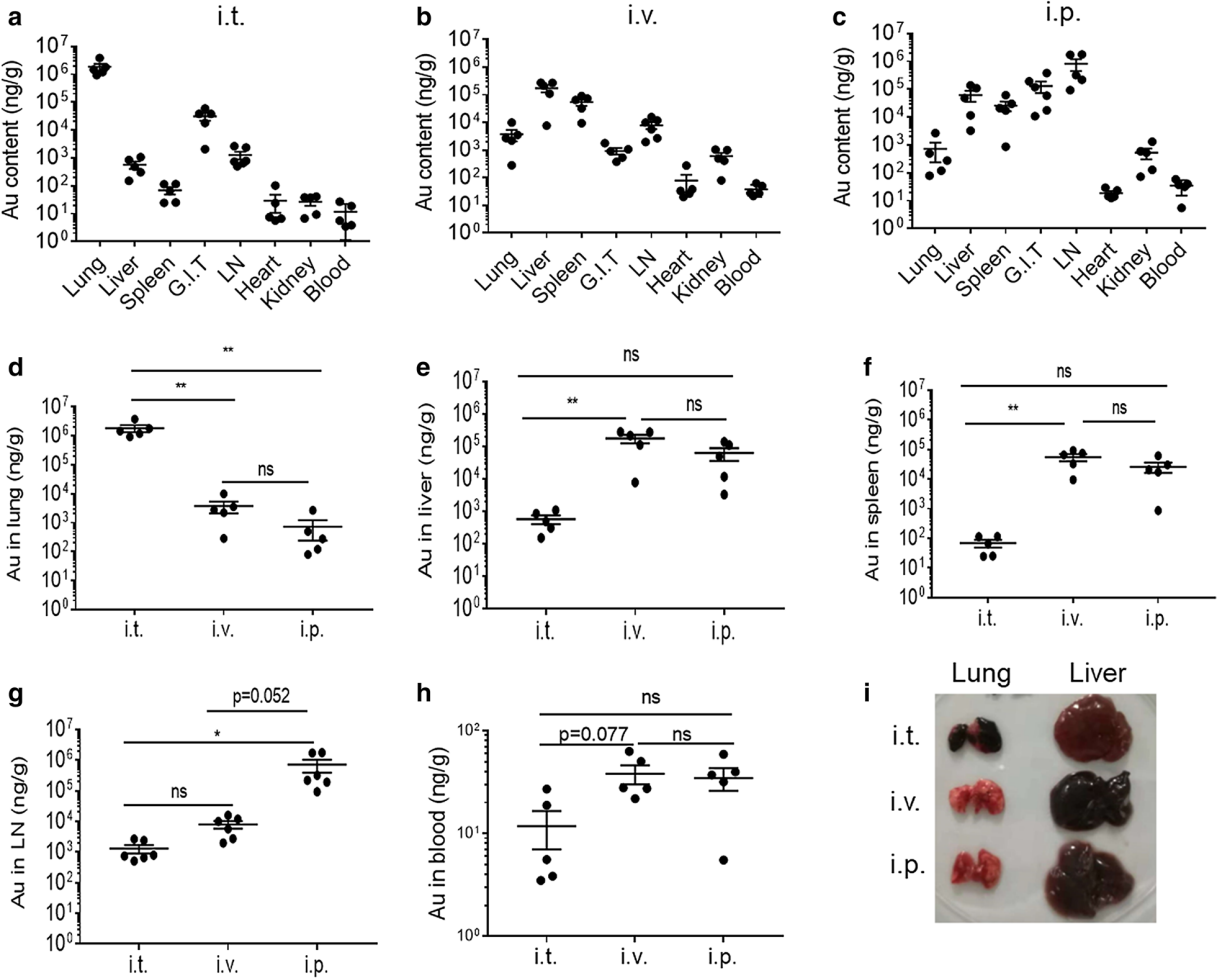
The biodistribution profile of P12 through i.t., i.p. and i.v. administration in healthy mice. Healthy mice were treated with P12-Cy5 (500 nM) for 4 h via three different administration routes: i.t. (**a**), i.v. (**b**) and i.p. (**c**), and the major organs/tissues were assessed by gold content (ng/g organ or tissue) using ICP-MS. The amount of gold in the lung (**d**), liver (**e**), spleen (**f**), LN (**g**) and blood (**h**) through different administration routes was analyzed. (**i**) The photograph of lung and liver for P12 accumulation (dark color) with different administration routes. N = 5 per group, ns: not significant, *p < 0.05, ** p < 0.01

In order to investigate the biodistribution of P12 in vivo affected by the different administration routes, we first studied the biodistribution profiles of P12 in healthy mice at an early time point of 4 h post P12 treatment. P12 was labeled with tiny amount of the fluorescence probe Cy5 (1%) (P12-Cy5) to allow for dual modules of analysis: in vivo fluorescence imaging and ICP-MS analysis. It was found that with i.t. administration, the accumulation of P12 in various organs/tissues from high to low followed the trend: lung >  > gastrointestinal tract (G.I.T) > lymph node (LN) > liver > spleen > kidneys ≈ heart ≈ blood (Fig. 5a); however, the trend was different with i.v. or i.p. route: liver > spleen > LN > lung > G.I.T > kidneys > heart ≈ blood (for i.v.) (Fig. 5b) and LN > G.I.T > liver > spleen > lung > kidneys > heart ≈ blood (for i.p.) (Fig. 5c). To better compare the biodistribution of P12 among the three administration routes, the accumulation of P12 in each organ/tissue was analyzed individually. It was clearly seen that the i.t. administration of P12 had the highest accumulation in the lung (Fig. 5d) while P12 with i.v. administration tended to accumulate in liver and spleen (Fig. 5e and f); interestingly, the i.p. administration of P12 resulted in the highest accumulation in the LN (Fig. 5g). The P12 level in blood showed no big differences among the three administration routes (Fig. 5h). Overall, these results showed that P12 was preferably accumulated in the lung and less in the liver through i.t. administration comparing to i.v. or i.p. administration, which could also been observed directly from the organ photograph (Fig. 5i), where the dark color of the organs indicated the accumulation of P12.

Taken together, these results suggested that different administration routes could have distinct biodistribution profiles. The P12 given by i.t. accumulated the most in the lung but much less in the liver and spleen. On the other hand, the i.v. administration led to higher accumulation of P12 in the liver and spleen, whereas the i.p. administration of P12 had preferred accumulation in the LN.

### Biodistribution profiles of P12 vs. PEG-modified GNPs in healthy mice through i.t. administration

**Fig. 6:**
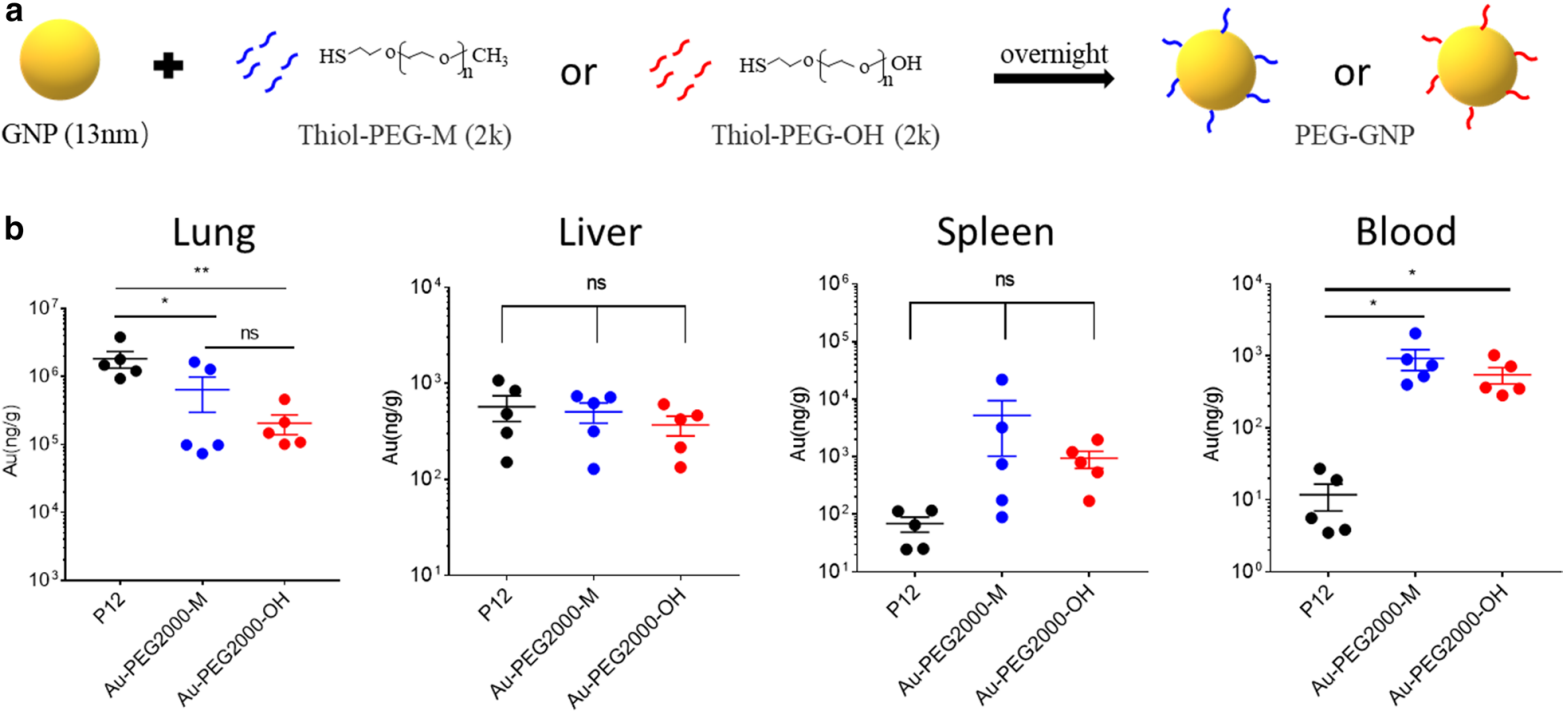
The biodistribution of PEGylated-GNPs in healthy mice through i.t. administration. **a** A diagram of the fabrication process of two types of PEGylated-GNPs. **b** The distribution of different PEGylated-GNPs in the lung, liver, spleen and blood. N = 5 per group, ns: not significant, *p < 0.05, ** p < 0.01

Based on the above results, the i.t. route appeared to have better therapeutic activity for P12 and an ideal biodistribution profile with P12 accumulated more in the lung than in the RES organs. However, we were wondering whether the favorable biodistribution profile of P12 by i.t. administration is P12 specific or due to the local administration only. To answer this question, we formulated two types of polyethylene glycol (PEG)-modified GNPs to replace the peptides of P12, and investigated their distribution in the lung, liver, spleen and blood at an earlier time point (4 h) and a later time point (24 h) after i.t. administration in the healthy mice. The two types of PEG ligands used were HS-PEG2000-M and HS-PEG2000-OH with a methyl group and a hydroxyl group at the end, respectively (Fig. 6a). These PEG-GNPs were formulated the same way as the P12 (Fig. 1a). At 4 h after i.t. administration, the amount of P12 in the lung was significantly higher than that of the Au-PEG2000-M and Au-PEG2000-OH (Fig. 6b). However, this difference disappeared at 24 h post treatment (Additional file 1: Fig. S1). There was no difference in the accumulation between P12 and the two PEG-modified GNPs groups in the liver and spleen at both 4 and 24 h post treatment (Fig. 6b and Additional file 1: Fig. S1). Interestingly, the P12 level in the blood was significantly lower than PEG-modified GNPs, suggesting that P12 was less capable of entering blood stream from the lung in comparison with PEG-modified GNPs. The two different end groups of PEG-GNPs (Au-PEG2000-M vs. Au-PEG2000-OH) had no effects on their biodistribution profiles. These results suggested that it was the unique characteristics of P12 that contributed to the preferred biodistribution of P12 in the lung in addition to the i.t. administration. Moreover, P12 seemed to be less efficient to cross the alveolar-blood barrier to enter circulation system than the PEG-GNPs.

We also compared the tissue biodistribution of P12 in healthy mice at 4 h and 24 h post i.t. administration. As shown in Additional file 1: Fig. S2, the P12 level in the lung was significantly decreased at 24 h, while its level in the spleen was increased. Nevertheless, the P12 content in the spleen via i.t. route was much less than that from the other two routes (Fig. 5f).

### The differential biodistribution profile of P12 in healthy and ALI mice upon i.t. administration

**Fig. 7:**
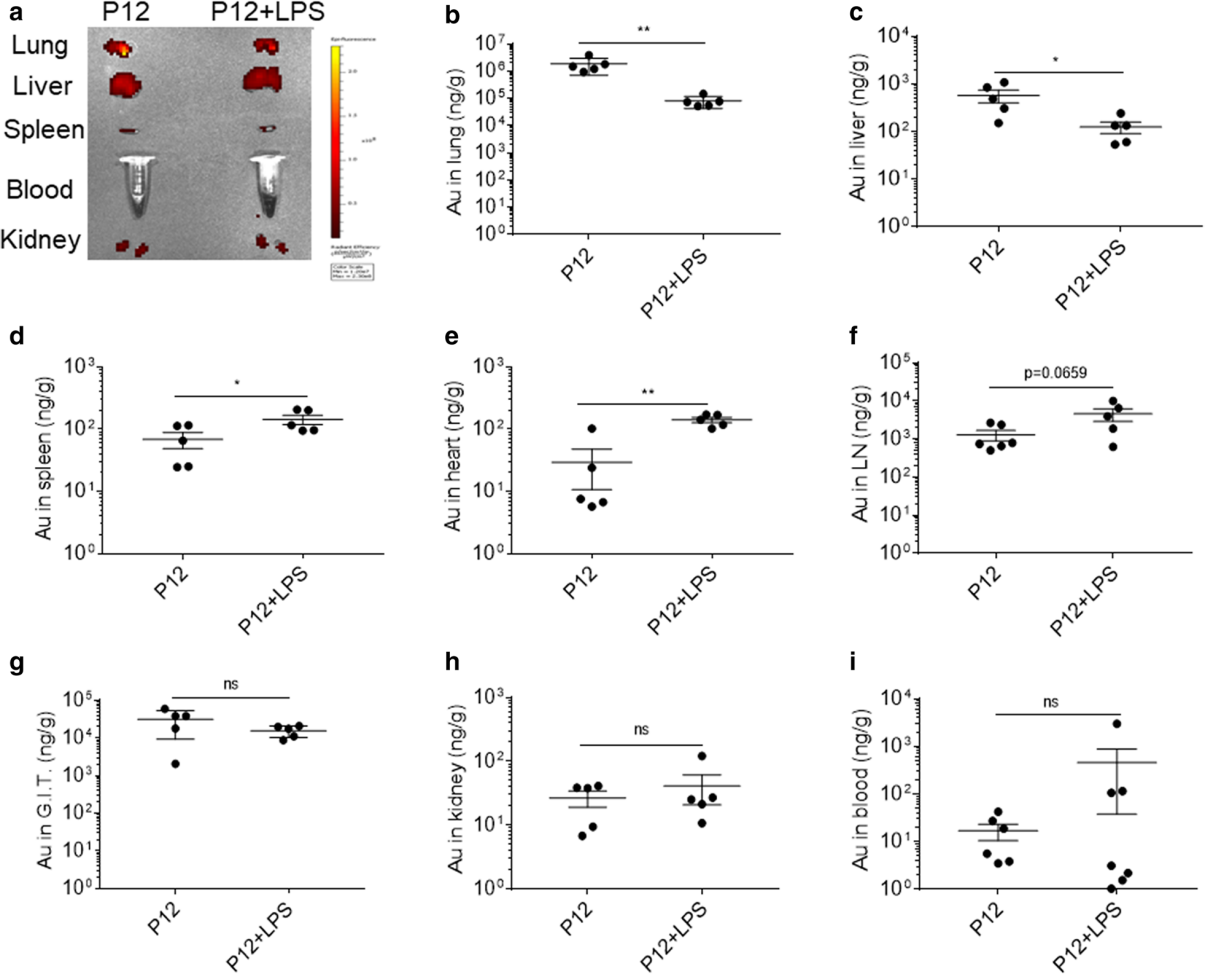
Comparison of the biodistribution of P12 through i.t. administration between healthy and LPS-induced ALI mice. P12-Cy5 was administered 2 h after LPS challenge, and the organs/tissues were collected 4 h after P12-Cy5 treatment. **a** A representative ex vivo image showing the fluorescence signals of P12-Cy5 in the lung, liver, spleen, blood and kidney. The amount of gold (Au) in each organ was quantified by ICP-MS as gold mass (ng) per gram of tissue in the lung (**b**), liver (**c**), spleen (**d**), heart (**e**), LN (**f**), G.I.T. (**g**), kidney (**h**) and blood (**i**). N = 5 per group, ns: not significant, *p < 0.05, ** p < 0.01

To further investigate whether the pathological condition influences the biodistribution profile of P12, the fluorescent P12 (labelled with Cy5) was intratracheally administered with or without LPS challenge. The organs/tissues were collected 4 h after P12 treatment. Ex vivo fluorescence imaging showed that the accumulation of P12 in the lung and liver was slightly higher in the healthy group than the LPS treated group (P12 + LPS), while the fluorescence signals from the spleen, blood and kidney were similar between the two groups (Fig. 7a). The overall biodistribution profile of P12 (by ICP-MS) in various organs/tissues upon LPS challenge was shown Additional file 1: Fig. S3. Such a profile was similar to that of healthy condition, where the level of P12 was still the highest in the lung, followed by G.I.T. When comparing the Au content in individual organ/tissue between healthy and LPS treated group, we found that the Au level in the lung and liver was significantly reduced in the pathological condition (Fig. 7b and c). However, the LPS treatment would enhance the accumulation of P12 in the spleen and heart (Fig. 7d and e), but had no effects on the Au content in LN, G.I.T, kidney and blood (Fig. 7f–i). These observations suggested that the presence of acute lung inflammation indeed affected the biodistribution of P12. The inflammatory condition in the lung promoted the distribution of P12 in the spleen and heart possibly through leaking from the alveoli to the blood vessel as the Au level in the blood seemed to be higher upon LPS challenge (Fig. 7i). Note that with or without LPS treatment, the intratracheally instilled P12 still had the highest distribution in the lung among all other organs/tissues, indicating that local pulmonary delivery of P12 may be ideal to treat ALI.

### The i.t. administration route led P12 to specifically target pulmonary macrophages

**Fig. 8:**
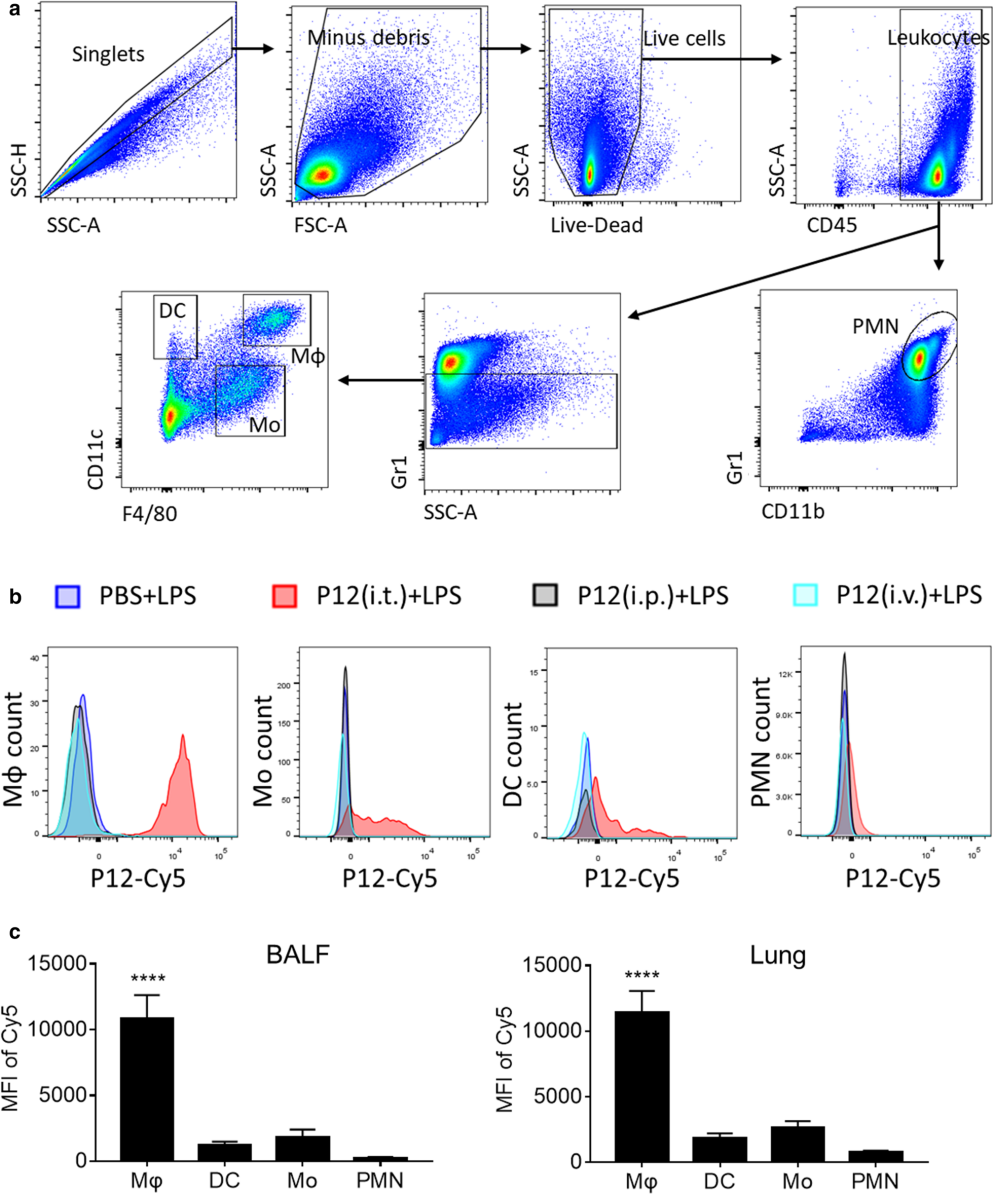
The i.t. administration of P12 targeting pulmonary macrophages. **a** Gating strategy of flow cytometry analysis to identify macrophages (MΦ), monocytes (Mo), dendritic cells (DC) and neutrophils (PMN) in the BALF and single cell suspensions of lung tissues. **b** Flow histogram of P12-Cy5 in the MΦ, Mo, DC and PMN from the BALF; P12 was administered by the three different routes. **c** The mean fluorescence intensity (MFI) of P12-Cy5 in the MΦ, Mo, DC and PMN from the BALF (left) and lung tissue (right); P12 was treated intratracheally. ****p < 0.0001 vs. all the other groups

Since i.t. administration of P12 showed better lung retention profile than i.v. and i.p. routes, it was expected that i.t. administration may enhance the targeting ability of P12 to pulmonary macrophages. To test this hypothesis, Cy5 labelled P12 pretreatment (2 h before LPS challenge) was given by i.t., i.v. or i.p. in a LPS-induced ALI mouse model. The infiltrated cells in the BALF and lung tissues were collected in the single cell suspensions at 24 h after LPS stimulation. Cells were stained with fluorochrome-conjugated antibodies to identify different phagocytic immune cells (Fig. 8a): macrophages (F4/80^+^CD11c^+^Gr1^−^), dendritic cells (F4/80^−^CD11c^+^Gr1^low^), monocytes (F4/80^low^CD11c^−^Gr1^low^) and neutrophils (Gr1^high^CD11b^+^) [24]. The flow histogram of Cy5-labeled P12 (P12-Cy5) in the above phagocytic cells collected from different administration groups showed that the fluorescence intensity of P12-Cy5 increased in all phagocytic cells only in the i.t. group, while that in the i.p. and i.v. groups were similar to the control (PBS + LPS) (Fig. 8b); the fluorescence signals were particularly stronger in the macrophages, indicating the macrophage targeting capability of P12. It should be noted that the uptake of P12-Cy5 by the phagocytic immune cells in the lung was minimum for i.v. and i.p. injection. Further analysis of the mean fluorescence intensity (MFI) of Cy5 among different types of immune cells for the i.t. group demonstrated that the uptake of P12 was primarily by macrophages in the BALF and lung tissues (Fig. 8c). These observations suggested that the i.t. route led P12 to be largely accumulated in the lung and specifically target pulmonary macrophages, while the other two administration routes could not.

## Discussion

The development of potent, targeted treatments to reduce the overwhelming inflammatory reactions in the lung and re-establish the homeostasis without harming other organs is still an unsolved challenge for many acute and chronic respiratory diseases. This study demonstrated that using the miniscule anti-inflammatory nanoparticles together with appropriate administration route could help overcome this challenge. First, we have found that the i.t. administration of nanodrugs was more efficient in managing lung inflammation than i.v. and i.p. administration routes. Second, the i.t. route also enabled nanodrugs to specifically target the effector inflammatory cells (i.e., lung macrophages) in the lung to attenuate inflammation in the early stage. Third, the local i.t. instillation could enhance the bioavailability of nanodrugs in the lung and reduce potential systemic side effects. Our findings have provided evidence that the i.t. administration may be a more effective and safer route to treat lung diseases for certain nanodrugs.

### Local delivery of anti-inflammatory nanoparticles P12 is more efficient for treating ALI

To date, ALI/ARDS caused by uncontrolled inflammatory response in the lung is still a big problem in the critical care medicine due to the lack of effective pharmacological treatments. Accumulated knowledge in ALI/ARDS pathogenesis reveals that pulmonary macrophages have played a critical role in the initiation, development and progression of the disease. Thus, targeting macrophages and manipulating their inflammatory status would provide a promising strategy to manage ALI/ARDS. We previously identified a novel anti-inflammatory nanoparticle (P12) that was effective in inhibiting multiple TLR pathways in macrophages and reducing lung inflammation and injury in a LPS-induced ALI mouse model [16, 19] Note that neither the peptide ligands nor the gold nanoparticles alone had inhibitory activity on TLR signaling [16]. Here, we further investigated the effects of the three commonly used administration routes, i.t., i.v. and i.p., on the therapeutic efficacy of P12 in the ALI mouse model. By comparing the three routes, the i.t. administration of P12 appeared to be better in reducing lung inflammation and injury particularly at a lower concentration (125 nM) than either i.v. or i.p. administration (Figs. 2 and 4). This administration route dependent effect may be explained in the following. The i.v. injection of P12 into the blood stream can quickly dilute P12 by circulating it throughout the body, so that only a small fraction of P12 could reach to the lung; similarly, P12 given by the i.p. injection will be adsorbed in the peritoneal membrane and majority of them would enter the blood stream. Therefore, it requires a higher dose of P12 via i.v. and i.p. administration to be effective in treating ALI (Figs. 3 and 4). In contrast, the i.t. route allowed all P12 to directly enter the airway and act on the effector cells efficiently, and hence a lower dose of P12 would be enough to show the therapeutic efficacy for ALI. The higher effectiveness of i.t. administration in treating respiratory diseases was also demonstrated in an infection model. It has been found that the airway administration of a therapeutic antibody conferred greater protection than parenteral administration (including subcutaneous, i.p. and i.v. injection) in treating pneumonia caused by pseudomonas aeruginosa [25]. Moreover, the i.t. administration of P12 not only increased the therapeutic effectiveness, but also provided preferred biodistribution profile (higher accumulation in the lung but much less in liver and spleen) (Figs. 5 and 7) and better targeting activity to alveolar macrophages (Fig. 8). Therefore, the topical i.t. administration route is an ideal way for P12 to treat inflammatory lung diseases.

For treating the lung diseases, the direct pulmonary delivery of drugs provides several advantages. First, a relatively low dose of drugs can be used to reach equivalent therapeutic effect when compared with the systemic delivery to possibly reduce the systemic side effects. Second, it is more efficient for the drugs to act in the lung, which is true for inhaled bronchodilators to treat asthma and COPD [26, 27]. Third, drugs can bypass hepatic metabolism to retain their maximum activity at the target sites right after administration. However, there are also some biological barriers to overcome for pulmonary delivery of nanodrugs. Mucociliary escalator in the airway tract is an efficient means of clearing foreign particles (such as nanodrugs) in the lung. If the nanodrug can pass through the mucociliary clearance and deposit to the alveoli, the alveolar macrophages will also clear them out through phagocytosis/endocytosis. The mucus layer on top of the airway epithelium is another barrier to trap and prevent nanodrugs from reaching the target cells. Because P12 primarily targets macrophages to exert its anti-inflammatory activity with multiple mechanisms of action (e.g., TLR inhibition and macrophage polarization) [18], it would be ideal to adopt pulmonary delivery for P12-like nanodrugs to treat ALI/ARDS in the future.

### Pulmonary delivery of nanodevices has a distinct biodistribution profile

The biodistribution profile of a given drug is important for its therapeutic efficacy and safety. Although studies on the biodistribution of intratracheal and intravenous injection of PEGylated GNPs have been reported [28], our study herein presented a systematic comparison on the biodistribution of a therapeutic nanoparticle among i.t., i.v. and i.p. administration to establish the correlation between the biodistribution profiles and their therapeutic effectiveness. Our results demonstrated that the i.t. administration allowed P12 mainly to be accumulated in the lung, while i.v. and i.p. administration let P12 primarily target the liver and the lymph nodes, respectively (Fig. 5a-c). Such a favorable biodistribution of P12 via i.t. administration is probably why it is more effective than other routes of administration to reduce LPS-induced ALI (Figs. 2, 3, 4).

It should be mentioned that although the amount of P12 in the lung (dark color) seemed to be high at 4 h post administration (Fig. 5i), we found that P12 could be quickly excreted out of the ALI mice 24 h after administration at which only about 5% of the injected dose were remained in the lung; this value was further decreased to about 0.5% after one month [19]. Furthermore, P12 was not toxic to human and murine macrophages (i.e. THP-1 cell derived macrophages and BMDMs) at the concentration used in this study [16, 18], and no obvious in vivo toxicity was observed throughout the experiments. Currently, we are conducting a more thorough in vivo biosafety studies to provide more insights for its future translational potentials.

Although nanotherapeutics are known for their better tumor targeting capability to treat cancer, extensive studies have shown that nanoparticles can still largely accumulate in the liver because they are easy to be trapped by the mononuclear phagocytic system (MPS, also known as RES) [29]. Even with PEGylation strategy, there are still about 60% of GNPs trapped in the MPS organs like liver and spleen after i.v. injection [28]. Our data showed that P12 accumulated much more in the lung and much less in the liver, spleen and LN when administrated through the airway compared with the i.p. and i.v. routes (Figs. 5 and 7), suggesting that pulmonary delivery of P12 may have relatively lower hepatic toxicity and side effects while having a higher bioavailability in the lung, which is ideal to treat inflammatory lung diseases. On the other hand, P12 had favor accumulation in the liver through i.v. administration, which may be potentially used to treat inflammatory liver diseases, such as autoimmune hepatitis [30, 31]. By analogy, the higher concentration of P12 in LN through i.p. administration may be applicable for treating chest lymph node metastasis [32]. Taken together, the observed distinct biodistribution profiles of P12 with different administration routes expand the therapeutic application of P12 for different diseases.

## Conclusions

Based on our previous discovery of the unique anti-inflammatory nanodevice P12, we herein systematically compared the effects of three different administration routes (i.t., i.v. and i.p.) on the therapeutic activity, biodistribution and pulmonary cell targeting ability of P12. The therapeutic efficacy of P12 was affected by the administration routes in a concentration dependent manner using a LPS-induced ALI mouse model. The i.t. instillation of P12 was superior in reducing lung inflammation and injury than the other two routes even at a lower concentration of P12. The biodistribution analysis revealed that the i.t. administration led to higher accumulation of P12 in the lung but less in the liver; however, the i.v. and i.p. administration resulted in more P12 accumulation in the liver and lymph nodes, respectively. This distribution profile could be influenced by the inflammatory condition in the lung, and was also dependent on the unique surface chemistry of P12. Moreover, the i.t. administration allowed P12 to target lung macrophages while the other two administration routes did not. This study raised an important consideration of choosing an appropriate administration route for nanodrugs to function effectively. It will also facilitate the development of effective targeted nano-therapies for treating ALI/ARDS in the future.

## Supporting information available

Additional figures describe the biodistribution of PEGylated-GNPs in the lung, liver and spleen of healthy mice through i.t. instillation at 24 h post administration, the distribution of P12 in different organs at 4 h and 24 h after P12 treatment (i.t.), and the effect of inflammatory status (LPS pretreatment) on the in vivo distribution of P12 in different organs. These materials are available free of charge via the journal website.

## Supplementary Information

**Additional file1: Figure S1**. The biodistribution of PEGylated-GNPs in the lung, liver and spleen in healthy mice through i.t. instillation at 24 h post administration. N = 5 mice per group, ns: not significant.** Figure S2**. In vivo distribution of P12 in different organs at 4 h and 24 h after P12 treatment (i.t.). The amount of gold in the main organs was quantified by ICP-MS. N ≥ 3 mice per group.** Figure S3**. In vivo distribution of P12 in different organs after LPS pre-treatment for 2 h. The organs were collected 4 h after P12 treatment. The amount of gold in the main organs was quantified by ICP-MS. N = 5 mice per group.

## Data Availability

All data generated and analyzed during this research are included in this article.
