# Supplementary material for: Administration route governs the therapeutic efficacy, biodistribution and macrophage targeting of anti-inflammatory nanoparticles in the lung
Source: J Nanobiotechnology. 2021 Feb 25;19:56. doi: 10.1186/s12951-021-00803-w (PMC7905431; doi:10.1186/s12951-021-00803-w)
Supplement: Supplementary file 1 — Additional file1: Figure S1. The biodistribution of PEGylated-GNPs in the lung, liver and spleen in healthy mice through i.t. instillation at 24 h post administration. N = 5 mice per group, ns: not significant. Figure S2. In vivo distribution of P12 in different organs at 4 h and 24 h after P12 treatment (i.t.). The amount of gold in the main organs was quantified by ICP-MS. N ≥ 3 mice per group. Figure S3. In vivo distribution of P12 in different organs after LPS pre-treatment for 2 h. The organs were collected 4 h after P12 treatment. The amount of gold in the main organs was quantified by ICP-MS. N = 5 mice per group. [file 12951_2021_803_MOESM1_ESM.docx]

**Supporting Information for**

**Administration route governs the therapeutic efficacy, biodistribution and macrophage targeting of anti-inflammatory nanoparticles in the lung**

Lu Wang^1,†,#^, Yafei Rao^1,#^, Xiali Liu^1,#^, Liya Sun^2^, Jiameng Gong^2^, Huasheng Zhang^3^, Lei Shen^3^, Aihua Bao^1,*^, Hong Yang^2,4,*^

^1^ Department of Pulmonary and Critical Care Medicine, Shanghai General Hospital, Shanghai Jiao Tong University School of Medicine, Shanghai 201620, China

^2^ School of Biomedical Engineering, Tianjin Medical University, Tianjin 300070, China

^3^ Shanghai Institute of Immunology, Shanghai Jiaotong University School of Medicine, Shanghai 200025, China

^4^ The Province and Ministry Co-Sponsored Collaborative Innovation Center for Medical Epigenetics, Tianjin Medical University, Tianjin 300070, China

#These authors contribute equally to the work.

†Current address: Department of Critical Care Medicine, Zhongda Hospital, School of Medicine, Southeast University, Nanjing 210009, China

*Corresponding author: Professor Hong Yang; Dr. Aihua Bao

Email: [hongyang@tmu.edu.cn](mailto:hongyang@tmu.edu.cn); [hongyang36@gmail.com](mailto:hongyang36@gmail.com); aihuabao312@126.com

**List of Supplementary Figures**

**Figure S1.** The biodistribution of PEGylated-GNPs in the lung, liver and spleen in healthy mice through i.t. instillation at 24 h post administration. N = 5 mice per group, ns: not significant.

**Figure S2.** In vivo distribution of P12 in different organs at 4 h and 24 h after P12 treatment (i.t.). The amount of gold in the main organs was quantified by ICP-MS. N ≥ 3 mice per group.

**Figure S3.** In vivo distribution of P12 in different organs after LPS pre-treatment for 2 h. The organs were collected 4 h after P12 treatment. The amount of gold in the main organs was quantified by ICP-MS. N = 5 mice per group.


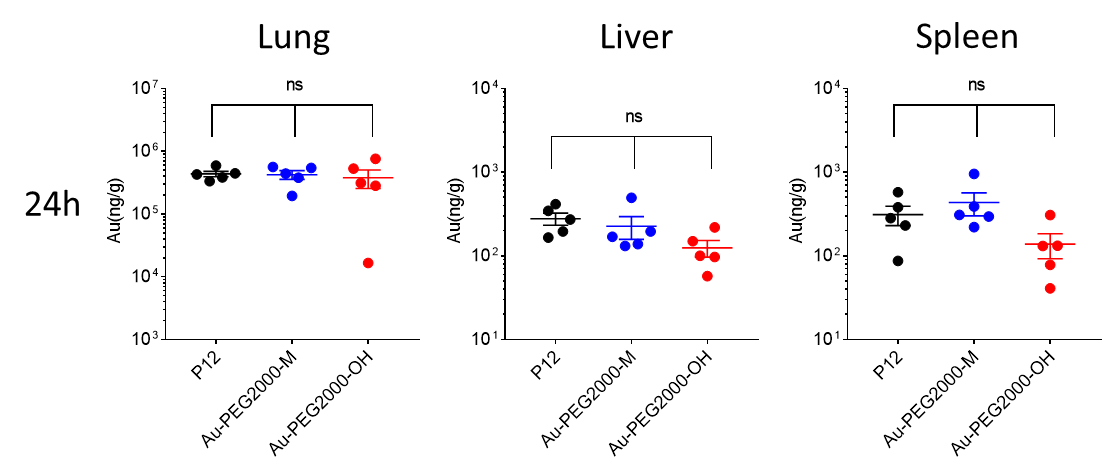


**Figure S1.** The biodistribution of PEGylated-GNPs in the lung, liver and spleen in healthy mice through i.t. instillation at 24 h post administration. N = 5 mice per group, ns: not significant.


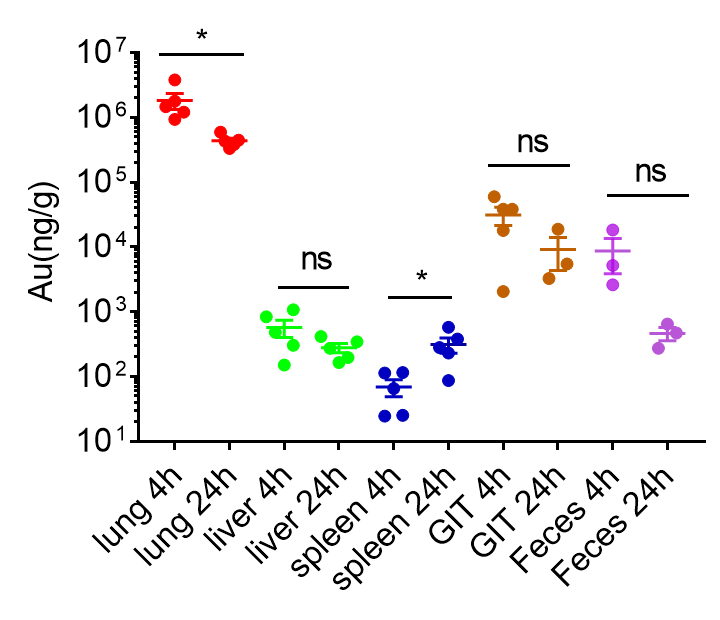


**Figure S2.** In vivo distribution of P12 in different organs at 4 h and 24 h after P12 treatment (i.t.). The amount of gold in the main organs was quantified by ICP-MS. N ≥ 3 mice per group.


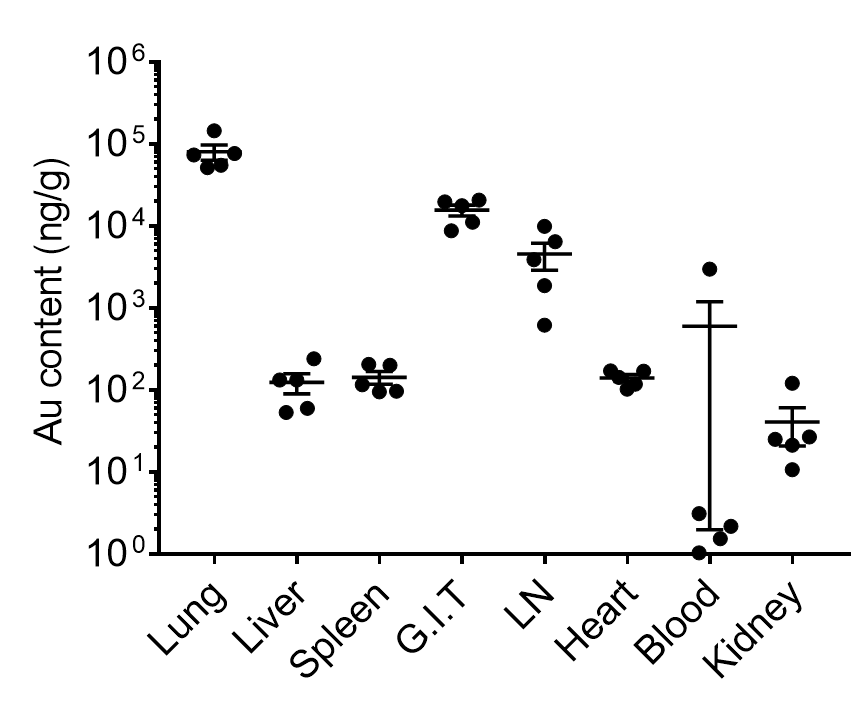


**Figure S3.** In vivo distribution of P12 in different organs after LPS pre-treatment for 2 h. The organs were collected 4 h after P12 treatment. The amount of gold in the main organs was quantified by ICP-MS. N = 5 mice per group.
